# P-2153. Ceftriaxone (CRO) pharmacokinetics (PK) in obesity in the intensive care unit (ICU)

**DOI:** 10.1093/ofid/ofaf695.2316

**Published:** 2026-01-11

**Authors:** Manasa Velagapudi, Catherine Palmer, Brent Inouye, Mara Wong, Nirpeksh Jain, Ahad Azeem, Rima El Herte

**Affiliations:** CHI Health - Creighton University Medical Center - Bergan Mercy, Omaha, Nebraska; Creighton University, OMaha, Nebraska; Creighton University, OMaha, Nebraska; Creighton University, OMaha, Nebraska; Creighton University School Of Medicine, Elkhorn, NE; Creighton University School of Medicine, Omaha, Nebraska; Creighton University School of Medicine, Omaha, Nebraska

## Abstract

**Background:**

CRO is a 3^rd^-generation cephalosporin used to treat various infections including bacteremia and meningitis. Several retrospective studies from ICU have documented that obese patients experience higher rates of treatment failure compared to non-obese patients receiving CRO. The purpose of this study was to compare serum ceftriaxone concentrations in obese (BMI ≥ 30 kg/m^2^) compared to non-obese patients (BMI < 30 kg/m^2^).

**Figure:**
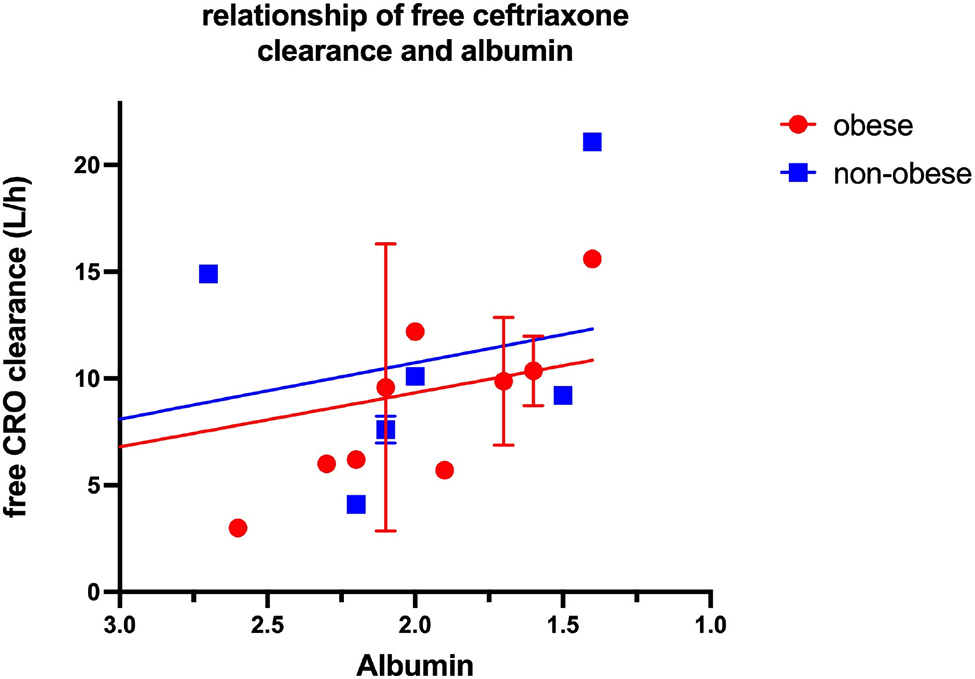
Relationship of free ceftriaxone clearance and albumin

**Methods:**

ICU patients (> 19 years of age) treated with CRO for a minimum of 48h were included. Pregnancy, transition to comfort care, CRO < 24h, and age < 19 years were excluded. All patients received 2000 mg IV q24h intravenous push as per hospital policy. Three blood samples (0.5h after the dose, midpoint, and 0.5h before the next dose) were obtained, allowed to clot, centrifuged, and serum frozen -80C until analyzed by LC-MS/MS for total CRO. Ultrafiltration using Amicon filter tubes were used for free CRO concentrations by LC-MS/MS. PK data were collected and analyzed using non-compartmental analysis with weighting 1/Y^2^ for area under the serum-concentration time curve (AUC_0-24_), clearance (CL), and half-life (t_1/2_). IRB approved this study (IRB #2003467) and patients or POA consented.

**Results:**

Eleven non-obese and 14 obese patients were consented and serum concentrations obtained for the two groups. No significant differences were found in patient age, height, qSOFA score, Crs, or albumin levels (obese 2.2 ± 0.6 vs. 2.5 ± 0.8 g/dl) between the two groups. Significantly higher weight and BMI in the obese group (obese 112. 7 ± 41.1 vs 67.2 ± 16.0 kg and 38.7 ± 11vs. 22.9 ± 4.4 kg/m2, p=0.002). Peak, trough and midpoint serum total and free CRO concentrations were not different between the two groups. For the >7 patients in each group who had albumin levels < 2.6 g/dl; free CRO clearance was significantly predicted by albumin concentration (Y=-2.5X +15.5 L/h), p< 0.05. Majority of patients (71%) had intra-abdominal infection that died. A total of 6/14 (43%) obese group died compared to 2/11 (18%) non-obese, p >0.05.

**Conclusion:**

ICU obese and non-obese patients did not clear CRO differently. Free CRO clearance was predicted by serum albumin if < 2.6 g/dl.

**Disclosures:**

All Authors: No reported disclosures

